# Shape-preserving erosion controlled by the graded microarchitecture of shark tooth enameloid

**DOI:** 10.1038/s41467-020-19739-0

**Published:** 2020-11-24

**Authors:** Shahrouz Amini, Hajar Razi, Ronald Seidel, Daniel Werner, William T. White, James C. Weaver, Mason N. Dean, Peter Fratzl

**Affiliations:** 1 Max Planck Institute of Colloids and Interfaces, Department of Biomaterials, 14476 Potsdam, Germany; 2 B CUBE - Center for Molecular Bioengineering, 01307 Dresden, Germany; 3 CSIRO Australian National Fish Collection, National Research Collections Australia, Hobart, TAS Australia; 4 School of Engineering and Applied Sciences, Harvard University, Cambridge, MA USA

**Keywords:** Biomechanics, Bioinspired materials, Biomineralization, Characterization and analytical techniques

## Abstract

The teeth of all vertebrates predominantly comprise the same materials, but their lifespans vary widely: in stark contrast to mammals, shark teeth are functional only for weeks, rather than decades, making lifelong durability largely irrelevant. However, their diets are diverse and often mechanically demanding, and as such, their teeth should maintain a functional morphology, even in the face of extremely high and potentially damaging contact stresses. Here, we reconcile the dilemma between the need for an operative tooth geometry and the unavoidable damage inherent to feeding on hard foods, demonstrating that the tooth cusps of Port Jackson sharks, hard-shelled prey specialists, possess unusual microarchitecture that controls tooth erosion in a way that maintains functional cusp shape. The graded architecture in the enameloid provokes a location-specific damage response, combining chipping of outer enameloid and smooth wear of inner enameloid to preserve an efficient shape for grasping hard prey. Our discovery provides experimental support for the dominant theory that multi-layered tooth enameloid facilitated evolutionary diversification of shark ecologies.

## Abstract (web summary)

Shark teeth have short lifespans yet can be subject to significant mechanical damage. Here, the authors report on a site-specific damage mechanism in shark teeth enameloid, which maintains tooth functional shape, providing experimental evidence that tooth architecture may have influenced the diversification of shark ecologies over evolution.

## Introduction

Teeth convert muscle contraction into contact force, in order to capture and mechanically process food^1–3^. The underlying dilemma is that stresses, which need to be large to break down food, challenge the teeth themselves. Since contact stresses increase as contact area decreases, sharper teeth apply higher stresses, but are also more prone to damage. Teeth, and especially their outer hypermineralized layers (enamel or enameloid, depending on species), have evolved to manage these extreme contact stresses, and as such are particularly relevant systems for understanding the coevolution of predators and hard-shelled or armored prey.

Across vertebrate taxa, the contact layer of teeth (i.e., enamel/enameloid) is comprised largely of the same basic components: a high percentage of calcium–phosphate mineral, and a small amount (<5%) of water and organic materials. In addition, despite some variation in the elemental composition of the inorganic phase (e.g., fluorine substitution in the teeth of sharks and some fishes^2,4,5^, or iron substitution in rodent incisors^6^), its composition remains relatively consistent across vertebrate taxa, consisting of nanocrystalline apatite^5,7^. These unifying material similarities across vertebrates are surprising, considering the diversity in the lifespan of vertebrate teeth^8^. For example, most mammals are diphyodont, with teeth replacement occurring only twice, but the majority of other vertebrates are polyphyodont, with teeth continuously grown and worn or serially replaced throughout the animal’s lifetime^3^. The form–function paradigms for contact materials in teeth, however, are largely distilled from the study of diphyodont mammals, whereas ~95% of vertebrates are polyphyodont. This heavily biases our understanding of the material origins of tooth contact performance, given that our models are based on systems where extreme durability is the rule, and where comparisons with other tooth types are rare.

Shark teeth represent an entirely different context for tooth materials: sharks exhibit a range of diets as functionally demanding as those of mammals, but produce, use, and discard teeth by the tens of thousands during their lifetimes. As a result, lifelong durability is irrelevant and their performance is only required for a few weeks rather than decades^9–11^. But if such differences in material lifespan are not reflected in tooth material composition, what role does microstructure play in the massively differential performance of vertebrate teeth?

**Fig. 1:**
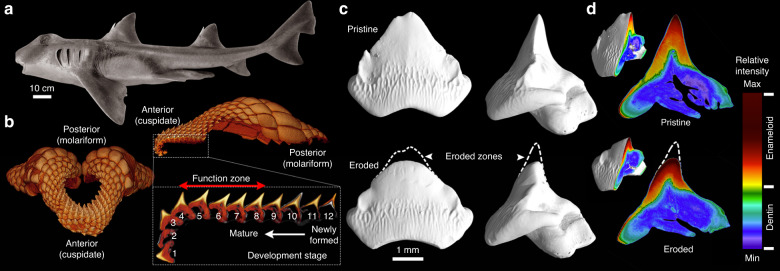
Anterior cuspidate teeth of the Port Jackson shark. **a** Photograph of an adult male Port Jackson shark (*H. portusjacksoni*). **b** X-ray microtomography image of the upper jaw tooth array showing the arrangement of the cuspidate and molariform teeth, at the anterior and posterior edges of the tooth array, respectively. The bottom inset image shows a single “file” of the cuspidate teeth, and the development from newly formed, to functional to post-functional teeth. **c** High-resolution X-ray microtomography demonstrating the change in tooth cusp morphology that occurs as teeth pass through the functional zone (i.e., from rows 8–4). **d** Virtual sections of the same pre-functional and post-functional teeth, illustrating erosion occurs only in the enameloid layer. In addition to the sharp transition in relative density between enameloid and dentin, a gradation in density is also present within the enameloid layer.

To answer these questions, in this study, we examine the links between microarchitecture and function in the grasping teeth of the Port Jackson shark (*Heterodontus portusjacksoni*; Fig. 1a). This species is from a family of sharks (the Heterodontidae), in which every species is ecologically and functionally specialized for consuming hard-shelled prey (durophagy). Unlike most other sharks, heterodontids specialize on shelled invertebrates (bivalves, crustaceans, etc.) and exhibit more than one tooth morphology (heterodonty), having pointed teeth in the front of the jaw for prey grasping, and flattened molars in the back for prey crushing (Fig. 1b)^12,13^. Many durophagous animals have molariform teeth for crushing, but the anterior pointed teeth of heterodontids are peculiar, since they are surely prone to extreme contact stresses, and mechanical erosion from grasping and feeding on hard-shelled foods, and from the grit associated with feeding in sandy and coralline environments. In particular, the incidental sediment and grit ingested as byproduct of a benthic ecology are likely to have a heavy influence on Port Jackson shark tooth wear, as illustrated by other taxa with microabrasive diets^14,15^.

In this work, we provide an in-depth analysis of the chemical, microscale and nanoscale structural composition, mechanical behavior, and damage mechanisms of the tooth enameloid of Port Jackson shark, providing an empirical performance test of enameloid tissue architectures thought to have been vital to shark dietary evolution. For this, we first characterize the enameloid microanatomy by light- and electron microscopy, as well as X-ray micro-computed tomography (μ-CT). We then use energy-dispersive X-ray spectroscopy (EDS) in the scanning electron microscope to show that the enameloid is chemically homogeneous. Based on field-emission scanning electron microscopy, as well as Raman microspectroscopic imaging, we then demonstrate and quantify an unusual graded microarchitecture of the enameloid. Finally, we show that although elastic modulus variations resulting from the graded enameloid microarchitecture do not have a decisive influence on the tooth’s mechanical performance, the local microarchitectures endow teeth with fracture and wear properties that lead to shape-preserving erosion.

## Results

### Micro-anatomical description of Port Jackson shark tooth enameloid

During their development, shark tooth “files” (anteroposterior columns of teeth; Fig. 1a) are continuously in motion, with individual teeth traveling from the dental epithelium inside the mouth, up and over the edge of the jaw, before being discarded after use^16^. Each tooth file, therefore, provides an instantaneous time series of progressive tooth development and maturation, including use and wear, as demonstrated, for example, in our X-ray µ-CT of jaws and teeth in the Port Jackson shark (Fig. 1b). The teeth currently in use (the functional teeth) are identifiable by their location in the jaw, but also by a change in morphology (Fig. 1b). Due to the change in orientation and location of tooth cusps in a file, only those paired with the opposite jaw teeth (i.e., rows 4–8) are in a position to contribute to grasping actions, whereas younger and older teeth (i.e. rows 9–12 and 1–3, respectively) are outside of the functional zone. Our µ-CT data show that functional and post-functional teeth exhibit clear abrasion and blunting, respectively, of the cusp (Fig. 1c, bottom row) when compared to the sharp and pointed pre-functional teeth (Fig. 1c, top row). This observation demonstrates that the cuspidate Port Jackson shark teeth, although likely not involved in the final crushing of prey, are indeed experiencing material damage during use. This provides also a material perspective on previous laboratory experiments, which showed a decrease in the performance of shark teeth with time, even when cutting softer foods^1^.

**Fig. 2:**
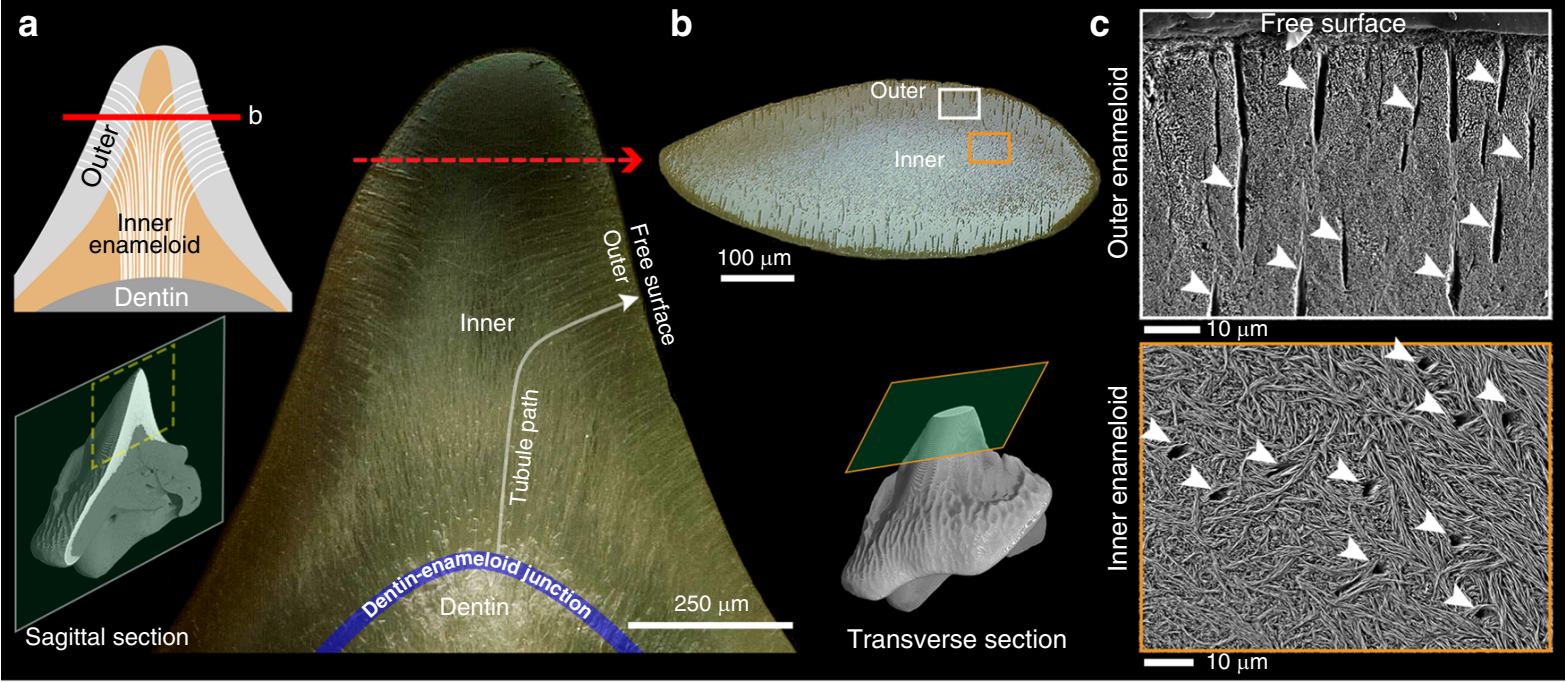
Microtubules in the enameloid layer of the Port Jackson shark teeth. **a** Dark-field optical micrograph of a sagittal section, and **b** bright-field optical micrograph of a transverse section of the cusp (rows 6–7) showing the paths of tubules from the inner toward the outer enameloid at the free surface of the cusp. **c** SEM micrographs, showing that the arced morphologies of microtubules result in their being cross- vs. longitudinally sectioned in the inner vs. outer enameloid, respectively (arrows).

Digital sections through µ-CT-scanned teeth allowed us to localize the damage caused by tooth wear to specific tissues (Fig. 1d). Unlike other shark teeth where dentin extends far into the cusp^17,18^, the cusp of Port Jackson shark teeth is comprised almost entirely of enameloid^19^. As a result, the observed erosion is limited to the enameloid layer and the underlying dentin is never exposed to contact. Optical and electron micrographs from cross-sectional planes of the cusp reveal a dense network of microtubules, coursing through the enameloid to end at the tooth surface (Fig. 2). The tubules appear to originate at the enameloid–dentin junction, and head predominantly toward the tooth tip yet turn sharply to orient perpendicular to the free surface in their last ~100 µm (Fig. 2). Tubules in dentin are a regular structural motif in vertebrate teeth, but enamel/enameloid tubules are far less well known, having been noted only sporadically in enamel of some reptiles and mammals (especially marsupials^20^), and in enameloid of both living and extinct elasmobranchs (sharks and rays)^21,22^. The function of enamel/enameloid tubules is unknown, in tooth mechanics or otherwise, but enamel tubules in mammals and reptiles are thought to perhaps convey the cell processes of enamel cells (ameloblasts) during tissue formation, and/or allow communication with odontoblasts in the underlying dentin^20,23^. Elasmobranch enameloid tubules are apparently an order of magnitude larger in diameter than their mammalian enamel counterparts^24,25^ and so may serve different roles, especially given that enameloid differs from enamel in being laid down by both ameloblasts and odontoblasts^17,26^.

In tooth biology, the modulus and mineral density difference between dentin and enameloid (or its mammalian functional analog, enamel) is believed to be a key factor in arresting cracks passing from one material to the other^27^. In Port Jackson shark teeth, in addition to the expected drop in X-ray attenuation levels and mineral density between enameloid and dentin, we also demonstrate a distinct gradient in X-ray attenuation within the enameloid layer, decreasing from the tooth surface toward the enameloid–dentin junction (Fig. 1d). This through-enameloid electron density gradient persisted even when teeth were scanned from different orientations (i.e., it was not a scanning artifact, such as from beam-hardening). Material gradients are important structural motifs in biological materials, used to build smooth structural bridges between tissues, but also for mediating mechanical function^28^. Demonstration of a material gradient in shark tooth enameloid reveals a distinct correspondence with mammalian teeth, where enamel exhibits a gradual and inverse change in modulus and toughness through its thickness^29^. While in mammals, this electron density gradient is believed to be a function of chemical changes through the enamel, related studies of shark teeth have never demonstrated similar trends^2^. Material density gradients can be functions of composition and/or structural property changes (e.g., degree of mineralization and/or porosity^30^), and in the following sections, we explore these options for Port Jackson shark teeth.

### The enameloid layer has uniform composition

**Fig. 3:**
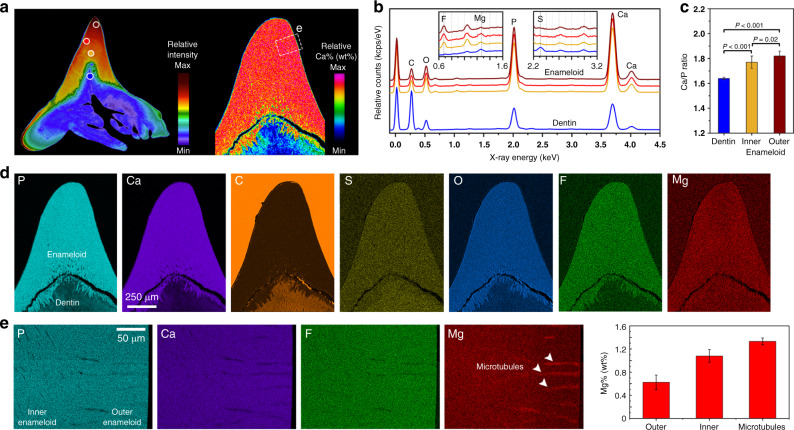
Elemental distribution maps extracted from sagittal section of the enameloid using high-resolution EDS analysis, showing the absence of site-specific compositional variation in the enameloid layer. **a** Spectral color code of Ca map (right) where higher calcium wt% is associated with higher relative intensity in µ-CT data (left and Fig. 1d). The locations of enameloid and dentin marked with circles were used for point elemental analysis studies. The colored dots indicate tooth regions (maroon: outer enameloid; red: outer-inner transition; orange: inner enameloid; and blue: dentin) are consistent also with **b** and **c**. **b** Comparison of the collected EDS data presenting the uniformity of the elemental spectra across different regions of the enameloid. **c** Mean values of Ca/P ratios showing a statistically significant (*P* < 0.001, *T* test: paired, *n* = 5) compositional shift between the dentin and both enameloid regions, whereas there was no significant difference (*P* = 0.02, *T* test: paired, *n* = 5) between the outer and inner enameloid (bars represent mean values ± standard deviations). **d** High-resolution EDS maps collected from the enameloid revealing a uniform distribution of the chemical elements. **e** EDS maps gathered with higher magnification and collected counts (longer probing time), revealing that microtubule walls contained the highest concentration of Mg (bars represent mean values ± standard deviations, *n* = 5).

Site-specific variations in chemical composition have been reported in a variety of biological structures—such as teeth, jaws, and appendages—that experience challenging loading environments. These gradients involve local variations in elemental density, but also inclusions of different materials. In chitons (*Cryptochiton stelleri*)^31^ or bloodworms (*Glycera dibranchiata*)^32^, for example, metals (zinc, iron) are incorporated into tooth or jaw materials, resulting in local performance enhancement of the tissues under severe contact stresses. To evaluate the presence of compositional heterogeneities in Port Jackson shark teeth, and to identify the chemical composition of the cusp, the elemental distribution of the enameloid was analyzed by EDS, using both point and map measurements (Fig. 3 and Supplementary Fig. 1). Port Jackson shark enameloid had high concentration of Ca, P, and F (Fig. 3), as also noted in previous investigations of shark teeth in other species^2^. Our data revealed that metallic trace elements were absent in the cusp, indicating that the enameloid is not locally reinforced by metals. Elemental distributions differed significantly between dentin and enameloid (Fig. 3b–d), whereas the inner and outer enameloid showed neither significant variation in elemental concentrations nor in Ca/P ratio. The latter has been correlated to the degree of mineralization in human enamel^33^. In Port Jackson shark teeth, we found that Ca/P ratios for the outer and inner enameloid were 1.82 ± 0.05 (wt%) and 1.77 ± 0.04 (wt%), and slightly lower than the reported Ca/P ratio for mature human enamel and dentin (~2.0 wt%)^34^. In contrast, the Port Jackson shark dentin layer possessed a significantly lower Ca/P ratio of 1.64 ± 0.01 wt%, comparable to human bone (~1.6 wt%). The subtle local variation in the Ca/P ratio, evidenced by assigning a spectral color to the Ca map (Fig. 3a), correlated with the intensity gradient seen in the µ-CT map (Figs. 3a and 1d). Moreover, the high-resolution EDS maps collected at higher magnification (Fig. 3e) revealed slight variations in Mg concentrations in the outer enameloid (0.63 ± 0.13 wt%), the inner enameloid (1.08 ± 0.11 wt%), and at the microtubule walls (1.33 ± 0.06 wt%). Mg can be used for phase stabilization of mineral precursors^35^.

### A graded microarchitecture of enameloid

**Fig. 4:**
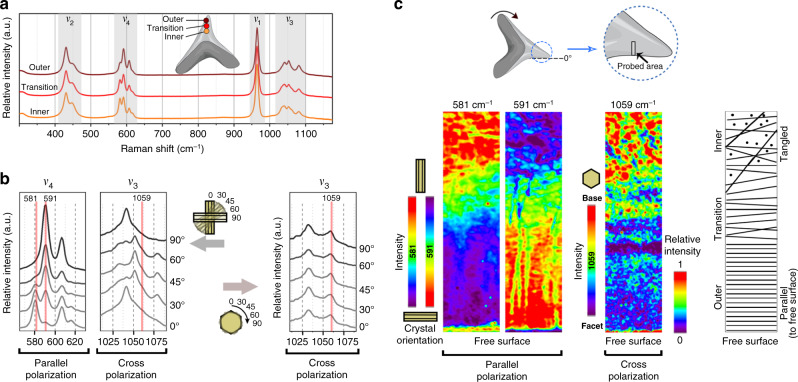
Raman analysis of the enameloid demonstrating the graded microarchitecture of the FAP crystallites. **a** Raman spectra profiles of enameloid regions (the color-coded spots marked on the tooth image), underlining that the enameloid is homogeneously crystalline fluorapatite in composition. The change in the relative intensity of vibrational band peaks *ν*_4_ (581 cm^−1^, 591 cm^−1^) and *ν*_3_ (1033 cm^−1^, 1041 cm^−1^), denoting variation in FAP crystallite alignment in different regions. **b** Polarized Raman spectroscopy study of a geological FAP monocrystal, used to calibrate the relationship between crystal orientation and peak intensity for *ν*_3_ and *ν*_4_ vibrational bands. Extracted spectra reveal that the relative intensities of the 581 cm^−1^ and 591 cm^−1^ peaks, as well as the presence of the 1059 cm^−1^ peak in cross-polarized measurements indicate the crystallographic orientations of the FAP crystals. **c** Collected Raman maps using parallel polarization and cross-polarization acquired in the rectangular ROI indicated in the schematic image at the top of the panel (scanning area of 50 μm × 194 μm). The maps were filtered for the orientation-sensitive peaks shown in **c** (581 cm^−1^, 591 cm^−1^, 1059 cm^−1^) to illustrate local variation in the relative peak intensity. By calibrating the collected maps from the enameloid according to the extracted spectra from the geological FAP (**b** and Supplementary Fig. 5), the orientations of FAP crystallites in different enameloid layers were quantified. As illustrated by the schematic image on the right of the panel, the parallel-aligned FAP crystallites at the free surface of the outer enameloid gradually transition to a tangled organization in the inner enameloid.

To investigate whether the material density differences in Port Jackson shark enameloid, observed in our high-resolution µ-CT data, could be also correlated with enameloid layer microstructure, we first used confocal Raman spectroscopy to determine the material composition of the enamel. The collected Raman spectra from different regions of the enameloid (Fig. 4a) showed a sharp *ν*_1_ vibrational band at 964 cm^−1^ (associated with a symmetric stretch mode of phosphate, PO_4_^3−^), indicating the enameloid layer is uniformly composed of crystalline fluorapatite (FAP; Supplementary Figs. 2 and 3)^36,37^. This composition was confirmed with X-ray diffraction measurements of the enameloid layer, showing clear peaks for (211), (300), and (202) lattice planes, indicating crystalline apatite (Supplementary Fig. 4).

Unlike many mineralized biological tissues, the high degree of mineralization of the enameloid layer resulted in a surprisingly large number of visible phosphate vibrational bands in the Raman spectra: 13 peaks from 4 vibrational bands (*ν*_1_–*ν*_4_; Supplementary Fig. 2a). Such an unadulterated, almost geological Raman mineral signal (i.e., such a high number of apatite phosphate vibrational bands) has never been reported in an apatite-based biomineralized tissue, even in highly mineralized, apatite-based biological tools (human teeth, mantis shrimp dactyl clubs, and parrotfish beaks). We took advantage of these strong phosphate vibrational bands, and how well they matched with reported phosphate peaks for a monocrystal of geological FAP^38^ to develop and calibrate a method for analysis of crystallographic arrangements in teeth.

First, we quantified relative changes in peak intensities of several FAP-relevant peaks (*ν*_4_: 581 cm^−1^, 591 cm^−1^; *ν*_3_: 1033 cm^−1^, 1041 cm^−1^), suggesting differences in the *c*-axis orientation of the FAP crystals between the inner and outer enameloid regions^38^ (Supplementary Fig. 2b). Accordingly, by mapping relative intensity variations using high-resolution confocal Raman imaging, filtering for relevant vibrational bands (*ν*_1_: 964 cm^−1^; *ν*_4_: 581 cm^−1^, 591 cm^−1^), changes in FAP crystallite orientation were suggested, moving from the free surface of the enameloid inward (Supplementary Fig. 2b).

However, conventional Raman spectroscopy does not allow discrimination of crystal orientations. To determine and visualize the arrangement of the FAP crystallites, we developed a nondestructive quantitative mapping method based on polarized Raman crystallography. With this technique, we again capitalized on the unveiled peaks of the phosphate (PO_4_^3−^) vibrational bands in Port Jackson shark enameloid and their similarity to those of geological FAP (Supplementary Note 1): by measuring Raman spectra from the basal and prism facets of a monocrystal of geological apatite in a variety of orientations, we generated orientation-dependent reference spectra for back determination of crystal orientation in enameloid.

**Fig. 5:**
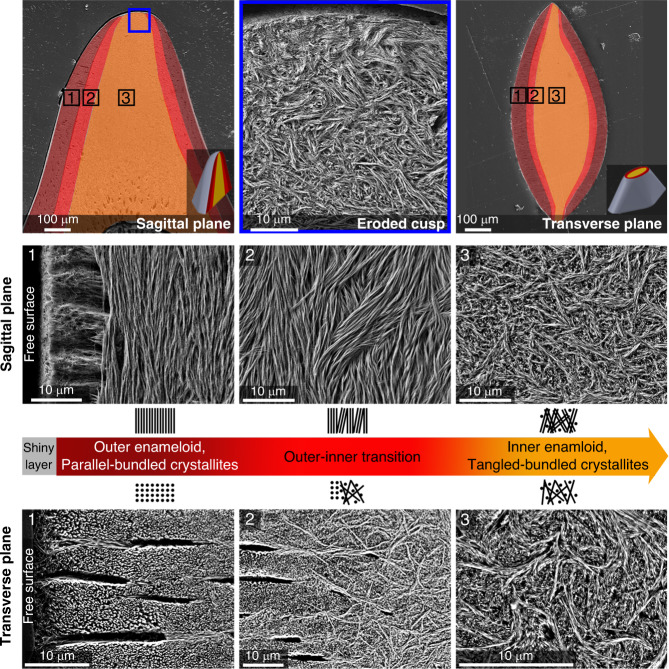
Graded microarchitectural arrangement of FAP crystallites in the enameloid layer. The FESEM micrographs collected from acid-etched sections of the enameloid reveal that while the outer enameloid is composed of parallel and aligned FAP bundles, the inner enameloid possesses a tangled and woven architecture. The inner enameloid at the apex of the cusp (top row, middle panel) is exposed by the natural erosion of the outer enameloid layer that occurs with use (e.g., from grasping hard prey, ingesting sediment). FESEM imaging also showed the thin (2–10 µm) shiny enameloid^63^ layer, comprising randomly packed crystallites and covering the outer surface of the tooth. The color designations for tooth regions are the same as in Fig. 3a–c.

This technique confirmed our hypothesis of a graded alignment of FAP crystallites in the cusp, wherein crystallites in the outer enameloid are aligned and parallel to the free surface, but gradually transition to a tangled organization in the inner enameloid (Fig. 4c). To verify this finding, we captured field-emission scanning electron microscopy (FESEM) micrographs from samples that had been etched chemically, an established technique for characterizing tooth crystallite organization. Our FESEM images supported our Raman data, showing a graded alignment of FAP crystallites in the cusp (Fig. 5). Our Raman approach therefore provided similar information to more traditional tooth crystallography techniques (i.e., ESEM imaging of acid-etched tissue), but is nondestructive, quantitative, and higher resolution, while also providing information on chemical composition (Fig. 5).

### Coexistence of crack guiding and deflection strategies promotes a shape-preservation mechanism

It is known that apatite crystals are stiffer along their *c*-axis^39,40^. Hence, a preferred alignment of the apatite crystallites toward the loading direction can promote stiffness in bioapatite-based mineralized tissues^37,41^. In contrast, woven and entangled microarchitectures are a common strategy for toughness enhancement in biomineralized tissues through promotion of crack deflection^5,42,43^. However, despite comprehensive recent work linking microarchitecture and mechanical properties of shark teeth^2,7,44^, to the best of our knowledge, there is no experimental evidence assessing the hierarchical interrelations of microarchitectural variations and multi-scale function of shark enameloid, particularly with regard to material performance and damage mechanisms in biologically relevant loading contexts.

Accordingly, we undertook a series of experiments to integrate material property data into macroscale finite element analysis (FEA) models of tooth performance. We first determined the micro-mechanical properties of the outer and inner enameloid in both dry and hydrated conditions, using nanoindentation studies of pristine (unworn) teeth (rows 7–8; Supplementary Fig. 6a, b). In the biting direction (transverse section), the outer enameloid possessed a higher elastic modulus and hardness (*E*_dry_ = 122.7 ± 3.5 GPa, *H*_dry_ = 4.6 ± 0.5 GPa) than the inner enameloid (*E*_dry_ = 97 ± 2.2 GPa, *H*_dry_ = 3.7 ± 0.7 GPa; Supplementary Fig. 6b). In the hydrated condition, the relationship was similar (outer enameloid: *E*_hyd_ = 93.8 ± 3.7 GPa, *H*_hyd_ = 2.2 ± 0.5 GPa; inner enameloid: *E*_hyd_ = 58.6 ± 5.3 GPa, *H*_hyd_ = 1.4 ± 0.2 GPa), but the contrast between inner and outer enameloid properties was more pronounced (Supplementary Fig. 6b). We conclude that such differentiation in elastic modulus and hardness of the outer and inner layer of the enameloid could be attributed to both packing and/or direction of the FAP crystallites^37^. When hydrated, while the mineral building blocks chiefly retain their rigidity, the organic phases soften further, accommodating shear strains and promoting sliding between FAP crystallites^45^, lowering the indentation elastic modulus^46^. The intracrystalline sliding mechanism is known as an effective mechanism for energy dissipation and toughening in biological ceramics^45,47^.

### Tooth geometry and applying damage to food

To consider the anatomical context of tooth performance, we captured the macroscopic geometry of tooth contact surfaces, in both pristine and eroded states. We used optical and electron micrographs to measure the contact curvature of pristine (row 8) and eroded teeth (row 3) in three different axes. The measured contact radii from the pristine and eroded enameloid (Supplementary Fig. 6c) showed that cusps exhibited hemi-ellipsoid geometries that were eroded from all contact directions, with the relative erosion (*r*_Eroded_/*r*_Pristine_) in the *z*-axis (the biting axis) being two times higher than in other directions (Supplementary Fig. 6c).

Accordingly, we integrated the extracted mechanical and geometrical data to assess the role of cusp stiffness, geometry, and the combination of both on stresses induced during contact with prey. We tested whether tooth material property or contact geometry is the dominant design criterion for inducing damage in prey/food. To do so, we designed two hypothetical tooth models: one composed purely of outer enameloid (*E* = 100 GPa) and one purely of inner enameloid (*E* = 60 GPa). For both material conditions, pristine and eroded cusp geometries were modeled and then the tooth applied to a crab shell with an elastic modulus of 600 MPa and yield modulus of 100 MPa^48^. We found that, independent of the modeled elastic moduli, the pristine models performed substantially better to initiate damage in the prey shell in comparison to eroded cusps (Supplementary Fig. 6d, dash lines versus solid lines). In order to damage the crab shell using a blunt cusp, the penetration depth had to be ~6 times higher in comparison to that with the pristine model, corresponding to a 34 times higher load (Supplementary Fig. 6f). This result reveals that independent of the elastic modulus, the contact geometry of the tooth plays a crucial role in damage initiation, illustrating the importance of maintaining tooth sharpness with regard to generating damage in ingested prey items.

Besides the microarchitecture and mechanical properties, the overall geometry can play a key role in the macro-mechanical response and performance of biological materials. For example, it has been shown that the subcritical curvature of mantis shrimp dactyl clubs prevents brittle fractures by promoting damage localizations through a quasi-plastic deformation^46^. The multi-cusp geometry of human molar teeth contributes to distribution of the damage zone and increases the load bearing capability of the tooth in comparison with a single-cusped tooth^49^. To obtain further insights into the mechanical response of the enameloid during the crushing action, we investigated how the enameloid material and the cusp geometry affect the functional response of the tooth under contact stresses.

### Tooth architecture and controlling damage to teeth

Despite our demonstration of enameloid ultrastructural layers with different material properties, our FEA models argue that in interactions with common hard-shelled prey items, enameloid modulus is not a decisive factor in tooth mechanical performance. Accordingly, we hypothesized that the graded microarchitectural arrangements in Port Jackson shark enameloid instead create a unique architecture for guiding damage, allowing the retention of cusp geometry. In support of this concept, it has been shown that tooth geometry and dimension could be a critical factor for its capacity in food manipulation, which defines the function of the tooth^50^.

**Fig. 6:**
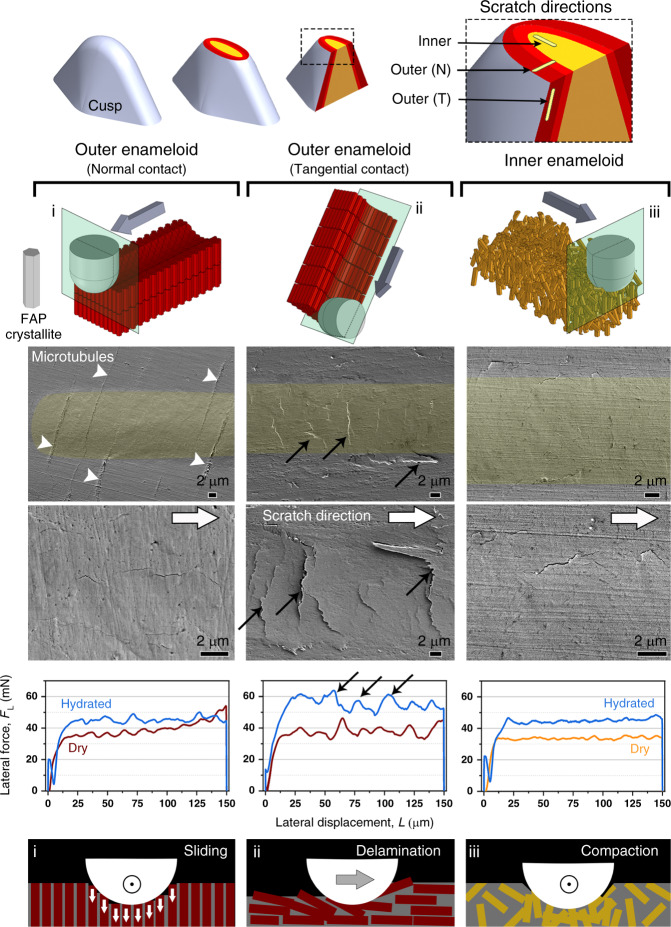
Scratch-induced damage mechanisms in the different layers of the enameloid. Post-scratch FESEM micrographs revealing the different induced damage modes, namely intercrystalline sliding (left column), delamination (middle column), and compaction (right column), occurring in different enameloid regions and under different loading orientations (also from left to right): in the outer layer under normal contact (biting direction), the outer layer under tangential contact (along the *c*-axis of FAP crystallites), and the inner layer. Scratching in the tangential orientation in the outer layer cause the most severe cracks and delamination across the scratch track, evidenced also by the force fluctuations in the lateral force-displacement curve (middle column). In hydrated conditions, despite an increment in lateral forces (due to higher contact depths), the pattern of the curves and the nature of the of the damages were consistent with the dry condition (Supplementary Fig. 7). In contrast, scratching in the normal orientation in the outer layer (left column) and inner layer (right column) induces only microcracks along the scratch tracks. The smooth erosion in the inner layer manifested by a wider scratch track and a smooth lateral force-displacement curve indicates compaction damage, which arises from the woven arrangement of the FAP crystallites and eventually prevents the formation of brittle damage. The color designations for tooth regions are the same as in Figs. 3a–c and 5.

To understand the inception and development of this damage in different enameloid layers, we designed a series of tribological studies simulating the interaction of the enameloid with hard targets, such as seashells, sea urchin spines and test, sand, and coral sediments^51^. First, we evaluated the mechanical response of the cusp under sliding forces using scratch tests in the inner and outer enameloid in the biting direction (normal to the transverse plane), and in the tangential direction (normal to the sagittal plane). The resultant damage revealed the response of crystallites to certain loading orientations and therefore the location-dependent mechanical behavior of enameloid. In the outer enameloid, loading in the biting direction promoted intercrystalline sliding, causing microcracks along scratch tracks (Fig. 6, left column), whereas tangential loading fostered delamination damage across scratch tracks (Fig. 6, middle column). As enameloid layers do not differ in their chemistry, our scratch tests argue that microarchitectural arrangements are solely responsible for regulating the mechanical response of the tooth cusp. By integrating these data with the crystallographic information from our developed Raman technique, we conclude that the outer enameloid microarchitecture provides predefined passages for crack propagation parallel to crystallite bundles. As a result, the demonstrated damage mechanisms indicate a preferred direction of crack evolution in outer enameloid; accordingly, we hypothesize that this crack guiding along the free surface promotes a crack deflection mechanism. This prescribed method of material loss is surprising, given that biological materials are often cited for their toughness and their tolerance to damage. In stark contrast, the scratch tests in the inner enameloid resulted in far smoother scratch tracks and finer microcracks with random orientations (Fig. 6, right column). This observation demonstrates the role of microarchitecture in the mechanics of the inner enameloid, where the tangled crystallite architecture promotes toughness through energy dissipation and crack deflection.

**Fig. 7:**
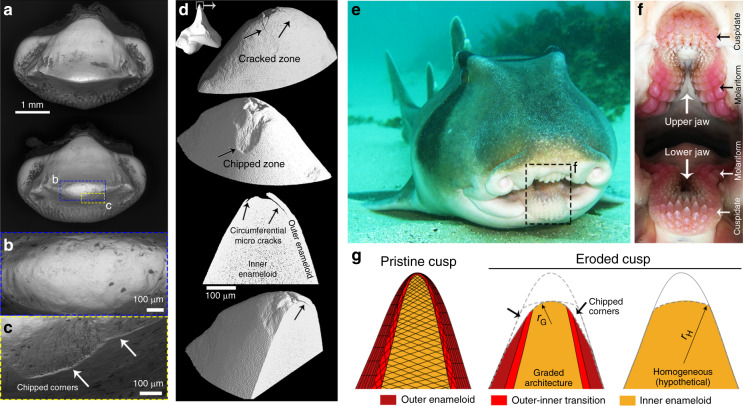
Differentiated damage modes in the outer and inner enameloid, likely induced by hard targets, such as seashells, sea urchin spines/test, sand, and sediments. **a**–**c** Wide-field backscattered scanning electron micrographs presenting **b** smooth erosion at the apex of the cusp and **c** chipped zones in the outer enameloid (white arrows). **d** High-resolution μ-CT images revealing the propagation of the circumferential microcracks beneath and along the free surface of the outer enameloid. These microcracks cause chipping damage. **e** Port Jackson shark photographs demonstrating the anterior grasping position of the cuspidate teeth, as points of first contact with food items. In the detail photo of the mouth (**f**), the teeth are red, believed to be from repeated ingestion of sea urchins, a common hard-shelled prey item (J. Kadar, pers. comm.). **g** A schematic illustration of the enameloid graded architecture and corresponding damage mechanisms. The graded microarchitecture of the enameloid layers results in chipping damage in the outer layer and smooth erosion in the inner layer, which promotes a higher rate of damage in the outer enameloid, and consequently a self-sharpening mechanism at the cusp. The estimated radii of an eroded cusp with a graded architecture (*r*_G_) and an eroded cusp with any given homogeneous architecture (*r*_H_) are illustrated for a rough visualization. The color designations for tooth regions are the same as in Figs. 3a–c and 5.

Our more macroscopic visualization methods support our hypothesis that local, nanostructural differences regulate specific controlled wear patterns in Port Jackson shark teeth. Using widefield scanning electron microscopy, we observed that in heavily used teeth (row 3), while the apex of the cusp was uniformly and smoothly eroded (Fig. 7b), the outer enameloid was partially chipped off (Fig. 7c). In contrast, the FESEM images (Supplementary Fig. 8) revealed that newer cusps (rows 8 and 9) lacked microcracks and chipping damage, confirming that the damage visualized in functional teeth had not been induced by dehydration. In a high-resolution μ-CT scan (isotropic voxel size of 345 nm) of a newly functional tooth (row 7), we determined the origin of the chipping damage to be circumferential microcracks propagated beneath and parallel to the free surface of the outer enameloid (Fig. 7d). In contrast, in our µ-CT and our microscopic studies (Figs. 2–4), the inner enameloid never exhibited obvious cracks, illustrating that peripheral damage is not propagated catastrophically through the cusp. These observations suggest that the inner enameloid accommodates induced damage in a non-brittle fashion, supported also by the smooth erosions visible on the exposed contact areas of inner enameloid in eroded teeth (Fig. 7b).

Taken together, our macroscale observations of tooth wear, tribological data, and damage analysis demonstrate differential damage mechanisms in outer and inner enameloid, with clear association to the tissue microarchitecture and resulting in a higher rate of damage to the outer enameloid. We argue below that this is a key factor in the prolongation of tooth performance despite—and, in fact, because of—material loss (Fig. 7g).

## Discussion

Nature has evolved a diversity of composite materials with a wide variety of properties and functions (both mechanical and ecological), despite having an extremely limited range of constituent materials to work with (minerals, proteins, lipids, and polysaccharides^52^). Damage-tolerant bioapatite-based ceramics involved in contact loading (e.g., teeth, dactyl clubs) represent extreme use cases for understanding how nature, through microarchitectural arrangement rather than chemistry, boosts mechanical performance of tissues (rigidity, toughness, and durability)^5,7,53^. Relying only on brittle apatite and scant, weak organic phases as building materials, these contact-loaded bioceramics play with ordered and disordered design strategies at multiple length scales to transcend the properties of their base materials, rendering impressive compound material properties. In human tooth enamel, for example, where crystallites are bundled into “rods” (arrays of apatite nanocrystals), the poor alignment of the crystallites within each rod promotes energy dissipation and fracture toughness^54^, whereas the co-alignment of apatite rods enhances hardness and stiffness in particular orientations^55^. Similarly, in mantis shrimp dactyl clubs, the misalignment and sliding of FAP crystallites promotes a quasi-plastic behavior^46^ and toughness^56^, important for the absorption of impact energy resulting from the smashing of mollusc shells. In the same system, the preferred orientation of FAP bundles toward the impact direction in the outer layer of the dactyl club enhances stiffness^37^. In parrotfish tooth enameloid, high toughness and abrasion resistance is fostered by an interwoven architecture of the FAP bundles^5^. Despite the different architectures of these bioceramic systems, in all cases, the uniformity of crystallite patterning through the entire contact layer helps to prolong the mechanical performance of the tissue.

Surprisingly, and in contrast with other contact-loaded bioceramics, Port Jackson shark tooth enameloid exhibits a microarchitectural inhomogeneity in the form of a graded arrangement of FAP crystallites, in which a parallel and aligned packing of the FAP crystallites in the outer enameloid shifts into an entangled arrangement in the inner enameloid. Accordingly, the enameloid manifests two distinct mechanical responses under the same contact stress regime, in which a crack-guiding mechanism promotes circumferential chipping damage in the outer enameloid, whereas a crack deflection strategy slows erosion by dissipating energy in the inner enameloid. Consequently, the coexistence of two damage mechanisms and the chipping behavior of the outer enameloid foster a functionalized damage mechanism that encourages nonuniform erosion of the tooth, resulting in a shape-preserving strategy that maintains cusp geometry, which is crucial for the tooth performance, as evidenced by our FEA analysis.

Other self-sharpening teeth (e.g., in sea urchins, rodents) also rely on chipping to maintain sharp edges, but the entire tooth tip experiences brittle fracture to promote material removal and create a fresh edge, while the tooth grows continuously, being pushed out from its base like a mechanical pencil’s lead. In contrast, the Port Jackson shark tooth can be likened to a pencil where new lead isn’t extruded, but rather the existing lead self-sharpens: peripheral chipping shapes the cusp, while the toughness provided by the inner enameloid prevents catastrophic failures, prolonging performance of individual teeth rather than relying on uninterrupted delivery of new material, where brittle fracture at the cusp of the tooth would greatly hinder functionality (similar to snapping the pencil’s lead).

Modern (extant) sharks exhibit a high diversity of different tooth morphologies, which are thought to be related to their respective feeding ecologies, from apex macropredators to filter-feeding giants, scavengers, and even parasites^16,57^. In the evolutionary radiation of modern sharks (crown Neoselachii), teeth became more ultrastructurally complex with regard to the layering of their enameloid, and it has often been posited that this structural differentiation was a key adaptive innovation in the evolutionary diversification of sharks and their feeding ecologies^26,58–62^. Our results provide experimental demonstration that shark tooth enameloid layers are indeed functionally differentiated, illustrating also that the integrated performance of the different layers grants important emergent properties to teeth at a more macroscopic level. These material strategies offer unique experimental support for prevailing theories of ecological radiation in sharks, but also insights for a new class of functional materials, attaining their performance by functionalizing damage through site-specific architectures, whereby material loss can be engineered and exploited for sustained high performance.

## Methods

### Sample collection

The teeth examined were from a specimen of *H. portusjacksoni* (field code #MUW058) from the personal collection of one of the authors (W.T.W), collected near Rottnest Island, Western Australia prior to July 2006 by commercial fishermen. The specimen was fixed and stored in 70% ETOH until being shipped to the Max Planck Institute for materials characterization. Teeth were carefully removed from the jaw for further processing.

### Sample preparation

For the light microscopy, electron microscopy, Raman spectroscopy, and indentation studies, the samples were embedded in an acrylic cold mounting resin (ClaroCit, Struers), sectioned using a diamond saw IsoMet 4000 (Buehler), ground with P4000-grade SiC paper, and finally polished with 1 and 0.25 μm colloidal solution. For the surface treatments used for electron microscopy purpose (Fig. 4c), the polished sections of the samples were etched with a solution of 8 M Urea and 5% acetic acid for ~8 min.

### X-ray micro-computed tomography

For µ-CT studies an X-ray microtomography scanner (Easytom, RX-Solutions), equipped with a micro-focus tube (XRay150, RX-Solutions) and a flat panel detector (CsI scintillator) was used. Scans were performed with a tube voltage of *U* = 85 kV, a tube current of *I* = 117 mA, an isometric voxel size of 7.37 µm, and an acquisition of *n* = 1120 filtered back projections in the continuous rotation mode (with reference images). Image stacks were reconstructed with a cone beam algorithm in the X-ACT software (RX-Solutions). 3D renderings and virtual sections were performed in Amira (Version 6.5, FEI). An additional μ-CT scan was performed of an entire dried tooth series of *H. portusjacksoni* from the British Museum of Natural History in London (BMNH 1867.4.2.59) to provide the overall anatomical perspective for Fig. 1b.

### Optical microscopy

Optical microscopic images were captured using a Leica digital microscope (DMV6, Country) in bright and dark-field modes.

### Field-emission scanning electron microscopy and energy-dispersive X-ray spectroscopy

Samples were imaged with a FESEM (JEOL JSM-7500F) at a low 5 kV accelerating voltage. To prevent surface charging, the samples were coated with 5–10 nm carbon and images were acquired with the lower secondary electron detector. EDS analysis was done on polished samples using an accelerating voltage 20 kV and emission current 10 μA, probe current 13 nA, and a live time of ~10,000 s.

### Wide-field scanning electron microscopy

Individual teeth were mounted to aluminum pin mounts using conductive carbon tape and examined in their native (uncoated) state using a Tescan (Brno, Czech Republic) Vega3 GMU variable pressure scanning electron microscope. All images were obtained using a large-area single-crystal YAG backscattered electron detector.

### Confocal Raman spectroscopy

A confocal Raman spectroscope (Alpha 300, Witec, Germany) equipped with a 532 nm laser wavelength and 20× magnification objective lens was used for the measurements. An integration time of 0.7 s and an accumulation time 90 s were used for acquisition in the point mode (single spectrum). Confocal mapping was done using an integration time 0.1 s and a 300 nm spatial resolution. To improve the signal-to-noise ratio and better detection of the peak positions, an 1800 grooves/mm grating was used for the measurements. The spectrometer was calibrated using a silicon wafer sample prior to the experiments.

For the polarized Raman measurements, an analyzer filter in 0° and 90° was used for the parallel and cross polarizations (the laser was filtered for 0°). For the measurements on the geological FAP crystal, the sample was rotated in different orientations of 0°, 30°, 45°, 60°, and 90°. The polarized maps (0° and 90°) from the shark enameloid were collected using an integration time 4 s and a 660 nm spatial resolution.

### X-ray diffraction measurement

X-ray diffraction measurements were conducted as a powder diffraction measurement on the powder sample. The powder was prepared from enameloids dissected from eight cusps and were crushed using a mortar. A Bruker AXS D8 ADVANCE instrument equipped with a Cu sealed X-ray tube producing Cu Kα-line with average wavelength, *λ* = 1.5418 Å, was used. X-ray diffraction profile was collected in the angular range, 2*θ* = 10°–55°, by using a scintillation detector.

### Mechanical characterization

Indentation studies were performed using a Triboindenter TI-950 nanoindenter (Hysitron-Bruker). The elastic modulus and hardness were measured using a standard transducer with 30 mN maximum load capacity and a 5 μm cono-spherical tip calibrated for the required range, using a standard fused quartz sample and a standard PMMA sample. For the experiments done in the hydrated conditions, the sample were immersed in water for 24 h prior to the experiment. A total number of 20–40 indents per region/condition were done for the measurements.

For the scratch tests, a 3D Omniprobe high-load transducer (Hysitron-Bruker) and a 50 μm fluid-cell cono-spherical tip was used. A normal load of 500 mN for length of 150 μm, and 30 s was set and used as scratch load function.

### Finite element modeling

3D finite element models of an eroded and a pristine cusp in contact with a crab shell were simulated using Abaqus (Dessault systemes, Simulia, RI). Contact curvatures and geometries were taken from CT scans of the two cusps and the shell. Surface-to-surface contact interaction was simulated between each two bodies (e.g., eroded cusp and shell), assuming a universal coefficient of friction of 0.2 in the tangential direction and a hard contact in the normal direction. Static loads were applied distally at the tooth with a ramp amplitude. In order to isolate the influence of geometry, identical elastic moduli of 60 and 100 GPa were applied to the bodies once. In the second set of analyses, eroded and pristine cusp contact models were compared using elastic moduli of 60 and 100 GPa, respectively.

### Data analysis

Data analysis and plotting were done using OriginPro software. Values are reported in mean ± SD format.

### Reporting summary

Further information on research design is available in the Nature Research Reporting Summary linked to this article.

## Supplementary information

Reporting Summary

Supplementary Information

## Data Availability

Data are available from the authors upon request.
