# Supplementary material for: Shape-preserving erosion controlled by the graded microarchitecture of shark tooth enameloid
Source: Nat Commun. 2020 Nov 24;11:5971. doi: 10.1038/s41467-020-19739-0 (PMC7686312; doi:10.1038/s41467-020-19739-0)
Supplement: Supplementary file 1 — Reporting Summary [file 41467_2020_19739_MOESM1_ESM.pdf]

nature research

Corresponding author(s): Peter Fratzl, Mason N. Dean

Last updated by author(s): Oct 19, 2020

Reporting Summary

Nature Research wishes to improve the reproducibility of the work that we publish. This form provides structure for consistency and transparency in reporting. For further information on Nature Research policies, see our Editorial Policies and the Editorial Policy Checklist.

Statistics

For all statistical analyses, confirm that the following items are present in the figure legend, table legend, main text, or Methods section.

n/a	Confirmed	
[ ]	[x]	The exact sample size (n) for each experimental group/condition, given as a discrete number and unit of measurement
[x]	[ ]	A statement on whether measurements were taken from distinct samples or whether the same sample was measured repeatedly
[ ]	[x]	The statistical test(s) used AND whether they are one- or two-sided<br>*Only common tests should be described solely by name; describe more complex techniques in the Methods section.*
[x]	[ ]	A description of all covariates tested
[x]	[ ]	A description of any assumptions or corrections, such as tests of normality and adjustment for multiple comparisons
[ ]	[x]	A full description of the statistical parameters including central tendency (e.g. means) or other basic estimates (e.g. regression coefficient) AND variation (e.g. standard deviation) or associated estimates of uncertainty (e.g. confidence intervals)
[x]	[ ]	For null hypothesis testing, the test statistic (e.g. F, t, r) with confidence intervals, effect sizes, degrees of freedom and P value noted<br>*Give P values as exact values whenever suitable.*
[x]	[ ]	For Bayesian analysis, information on the choice of priors and Markov chain Monte Carlo settings
[x]	[ ]	For hierarchical and complex designs, identification of the appropriate level for tests and full reporting of outcomes
[x]	[ ]	Estimates of effect sizes (e.g. Cohen's d, Pearson's r), indicating how they were calculated

Our web collection on statistics for biologists contains articles on many of the points above.

Software and code

Policy information about availability of computer code

Data collection	N.A.
Data analysis	N.A.

For manuscripts utilizing custom algorithms or software that are central to the research but not yet described in published literature, software must be made available to editors and reviewers. We strongly encourage code deposition in a community repository (e.g. GitHub). See the Nature Research guidelines for submitting code & software for further information.

Data

Policy information about availability of data

All manuscripts must include a data availability statement. This statement should provide the following information, where applicable:

- Accession codes, unique identifiers, or web links for publicly available datasets
- A list of figures that have associated raw data
- A description of any restrictions on data availability

Data are available from the authors upon request.

Field-specific reporting

Life sciences study design

All studies must disclose on these points even when the disclosure is negative.

Sample size	~20 individual teeth were used for the study, taken from comparable tooth files in the jaw.
Data exclusions	No data were excluded from the analysis.
Replication	The number of replicates varied by experiment and is mostly listed in the text. However, for all material characterization tests, 3-5 different samples were used to verify results. In the case of nanoindentation, 20-40 indents were performed per sample, as mentioned in text.
Randomization	For the purpose of our study, characterizing enameloid properties from mature teeth, randomization was not relevant, as our goal was to focus on one experimental group (i.e. mature teeth). The tooth number (representing maturity - see Fig. 1) used was consistent across experiments in the study.
Blinding	Blinding is also not relevant for our study, in that we were focused only on teeth of a particular maturity stage (see previous answer).

Reporting for specific materials, systems and methods

We require information from authors about some types of materials, experimental systems and methods used in many studies. Here, indicate whether each material, system or method listed is relevant to your study. If you are not sure if a list item applies to your research, read the appropriate section before selecting a response.

Materials & experimental systems

n/a	Involved in the study	
☒	☐	Antibodies
☒	☐	Eukaryotic cell lines
☒	☐	Palaeontology and archaeology
☐	☒	Animals and other organisms
☒	☐	Human research participants
☒	☐	Clinical data
☒	☐	Dual use research of concern

Methods

n/a	Involved in the study	
☒	☐	ChIP-seq
☒	☐	Flow cytometry
☒	☐	MRI-based neuroimaging

Animals and other organisms

Policy information about studies involving animals; ARRIVE guidelines recommended for reporting animal research

Laboratory animals	The study did not involved laboratory animals.
Wild animals	The specimen used came from a commercial fishery, see sample information in the following answer and in text.
Field-collected samples	The teeth examined were from a head specimen of H. portusjacksoni (field code #MUW058) from the personal collection of one of the authors (W. White), collected near Rottnest Island, Western Australia prior to July 2006 by commercial fishermen.
Ethics oversight	No ethical approval or guidance was required as the specimen was a discard from a commercial fishery.

Note that full information on the approval of the study protocol must also be provided in the manuscript.
