# Supplementary material for: Shape-preserving erosion controlled by the graded microarchitecture of shark tooth enameloid
Source: Nat Commun. 2020 Nov 24;11:5971. doi: 10.1038/s41467-020-19739-0 (PMC7686312; doi:10.1038/s41467-020-19739-0)
Supplement: Supplementary file 2 — Supplementary Information [file 41467_2020_19739_MOESM2_ESM.pdf]

Supplementary Information

Shape-Preserving Erosion Controlled by the Graded Microarchitecture of Shark Tooth Enameloid

Shahrouz Amini[1], Hajar Razi[1], Ronald Seidel[1,2], Daniel Werner[1], William T. White[3], James C. Weaver[4], Mason N. Dean[1*], Peter Fratzl[1*]

Affiliations:
1 Max Planck Institute of Colloids and Interfaces, Department of Biomaterials, 14476 Potsdam, Germany
2 B CUBE - Center for Molecular Bioengineering, 01307 Dresden, Germany
3 CSIRO Australian National Fish Collection, National Research Collections Australia, Hobart, Tasmania, Australia
4 Wyss Institute for Biologically Inspired Engineering, Harvard University, USA

* Corresponding authors: Peter.Fratzl@mpikg.mpg.de, Mason.Dean@mpikg.mpg.de

Supplementary Figures

Supplementary Fig. 1
Supplementary Fig. 2
Supplementary Fig. 3
Supplementary Fig. 4
Supplementary Fig. 5
Supplementary Fig. 6
Supplementary Fig. 7
Supplementary Fig. 8
Supplementary Fig. 9

Supplementary Note

Supplementary Note 1. Raman Crystallography

Supplementary Figures

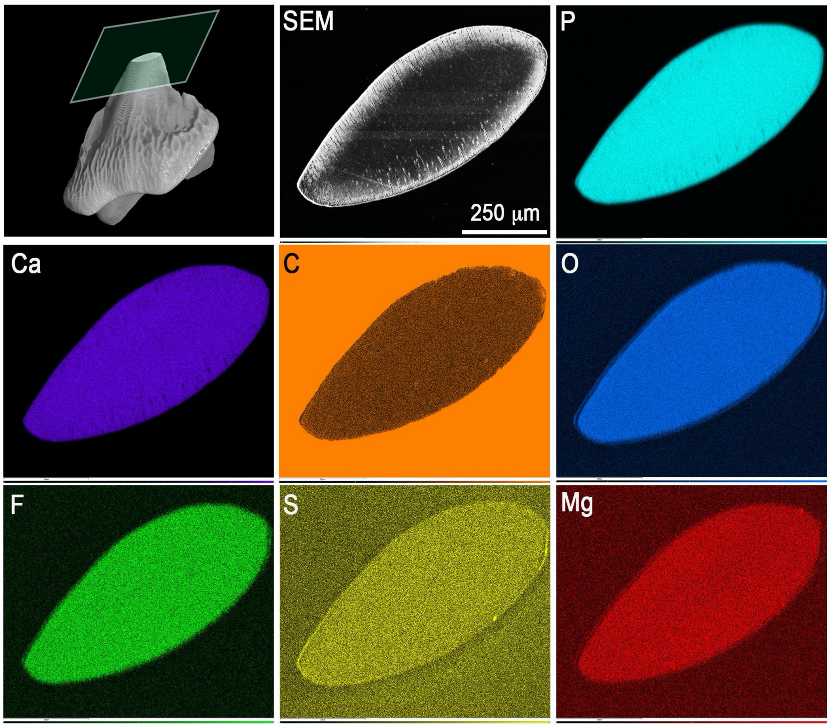

Supplementary Fig. 1 Elemental distribution maps extracted from a transverse section of the enameloid layer showing the absence of elemental distributions in the enameloid layer. The uniform distribution of the element was in conformity with the extracted maps form the transverse section of the cusp (Fig. 3).

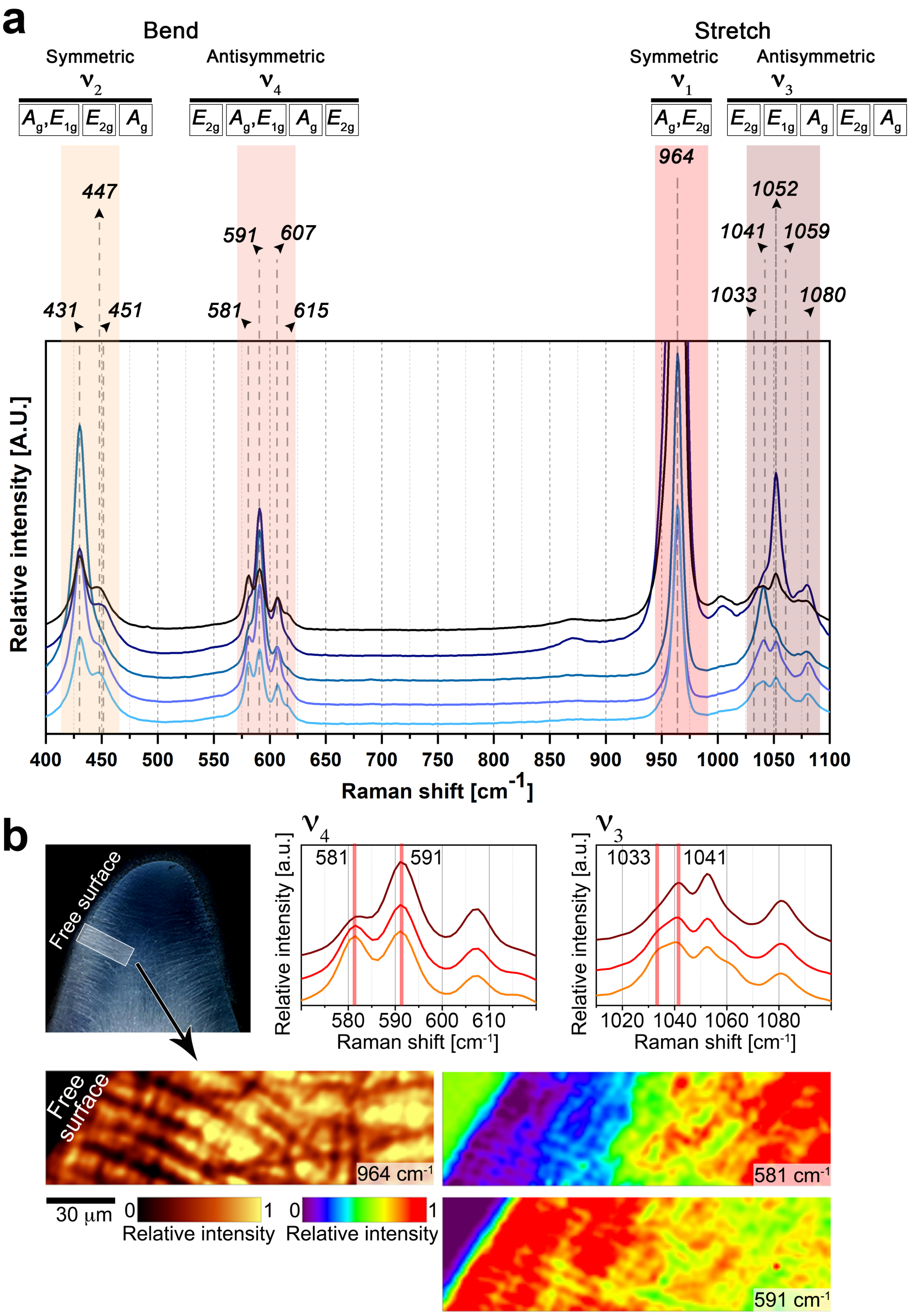

Supplementary Fig. 2 Raman spectra of the phosphate vibrational bands (ν_1 to ν_4) measured from the fluorapatite crystallites of the enameloid layer. **a** The visibility of the 13 peaks, which has been reported for a monocrystal of geological FAP[1], associated with the high degree of mineralization (low organic content) in the enameloid layer. **b** High-resolution Raman maps acquired in the correspondingly labeled box, and filtered for the peaks in vibrational bands ν_4 (581 cm^{-1}, 591 cm^{-1}), illustrating a graded alignment of crystallites in the enameloid.

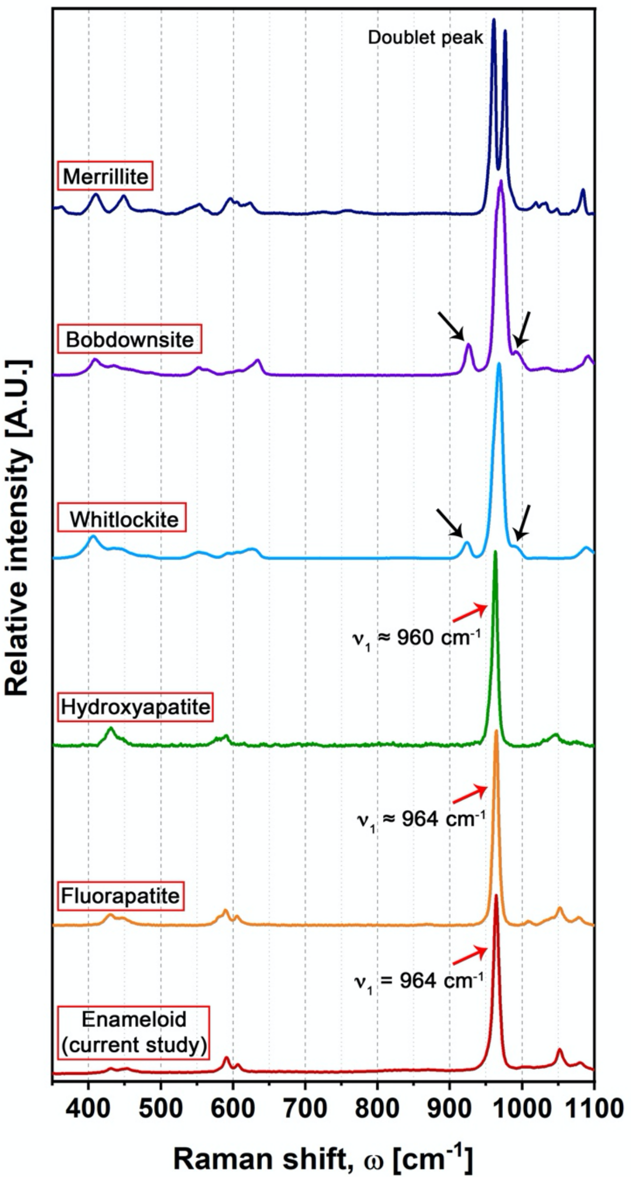

Supplementary Fig. 3 Raman spectra for various minerals, compared with that collected from the enameloid layer of Port Jackson shark teeth, revealing a match with fluorapatite (comparative Raman data extracted from the RUFF database: https://rruff.info). The Raman shift in the ν_1 peak of the phosphate band from ~960 cm^{-1} to ~964 cm^{-1} indicates the presence of apatite in its fluorinated form (fluorapatite) [2, 3].

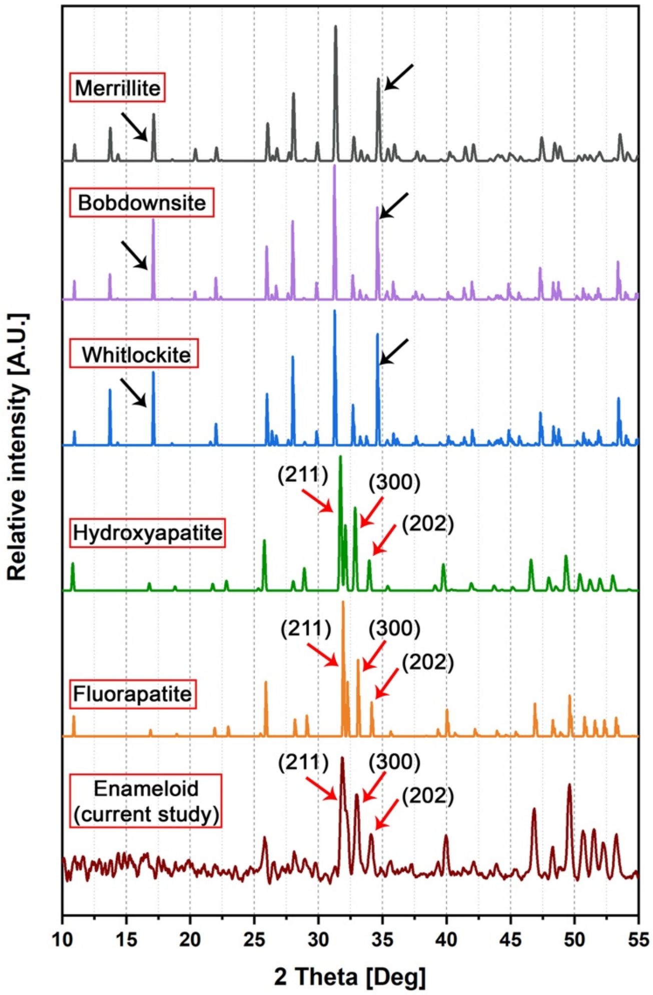

Supplementary Fig. 4 X-ray diffraction measurement on the enameloid of the teeth illustrating a perfect match with lattice parameters of apatite but not Whitlockite, Bobdownsite, or Merrillite (comparative XRD data extracted from the RUFF database: https://rruff.info). The differences of hydroxyapatite and fluorapatite are not easy to be distinguished using X-ray diffraction analysis [3].

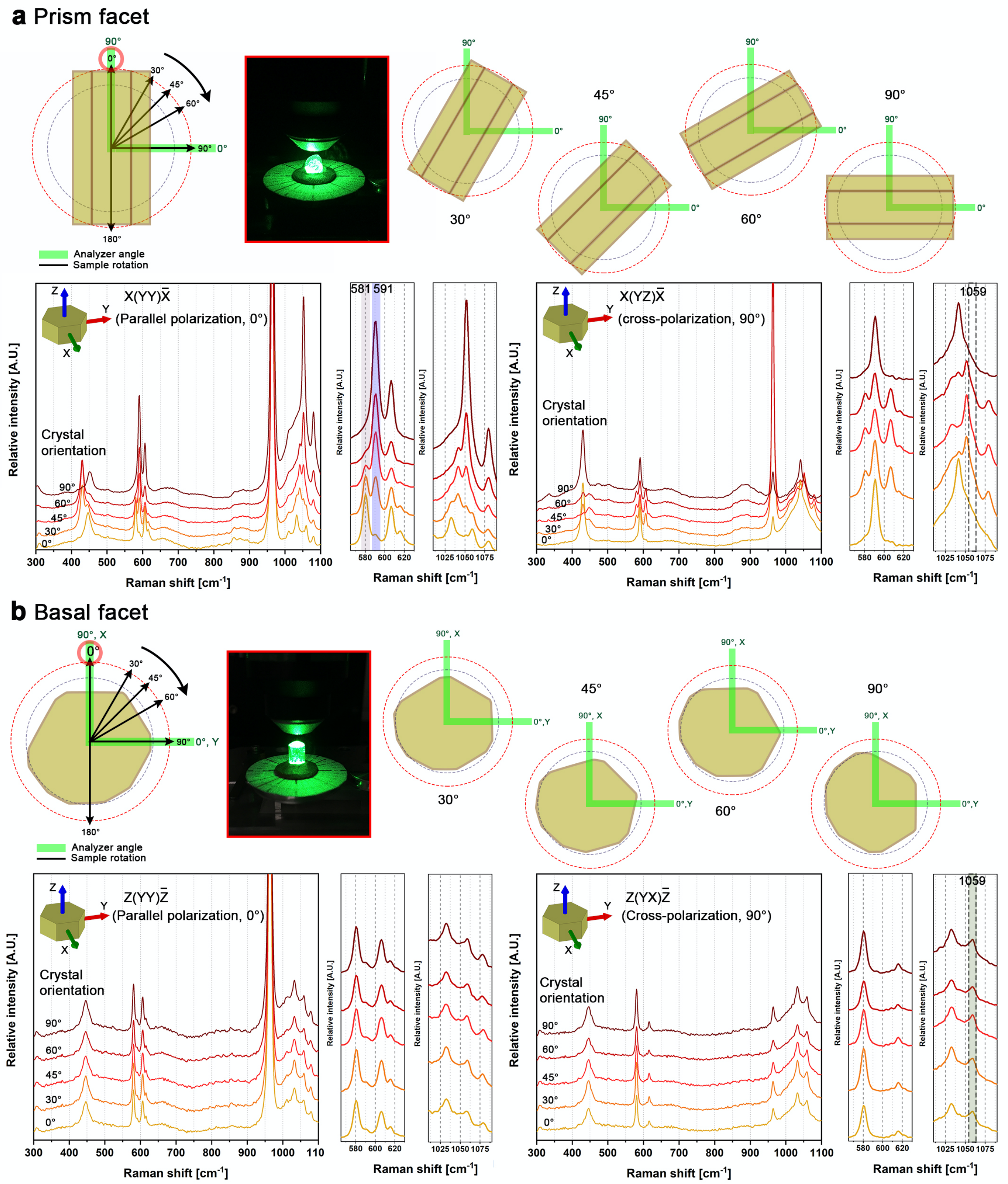

Supplementary Fig. 5 Polarized Raman spectroscopy on the monocrystal of geological FAP. The obtained spectra from **a** prism facet and **b** basal facet of the crystal in parallel and cross polarization along with rotation of the crystal in different orientations (0°,30°,45°, 60°, and 90°) representing the peak intensity-orientation dependencies. Accordingly, the three 581 cm^{-1}, 591 cm^{-1}, and 1059 cm^{-1} peaks were used for identification of the FAP crystal orientations. While the relative intensity of 581 cm^{-1} and 591 cm^{-1} peaks leads to identification of the prism facet and correlated orientations of c-axis (a), the presence of 1059 cm^{-1} peak at cross-polarization indicates the basal facet of FAP crystals (b). This peak is absent in the cross-polarized spectra extracted from the prism facet.

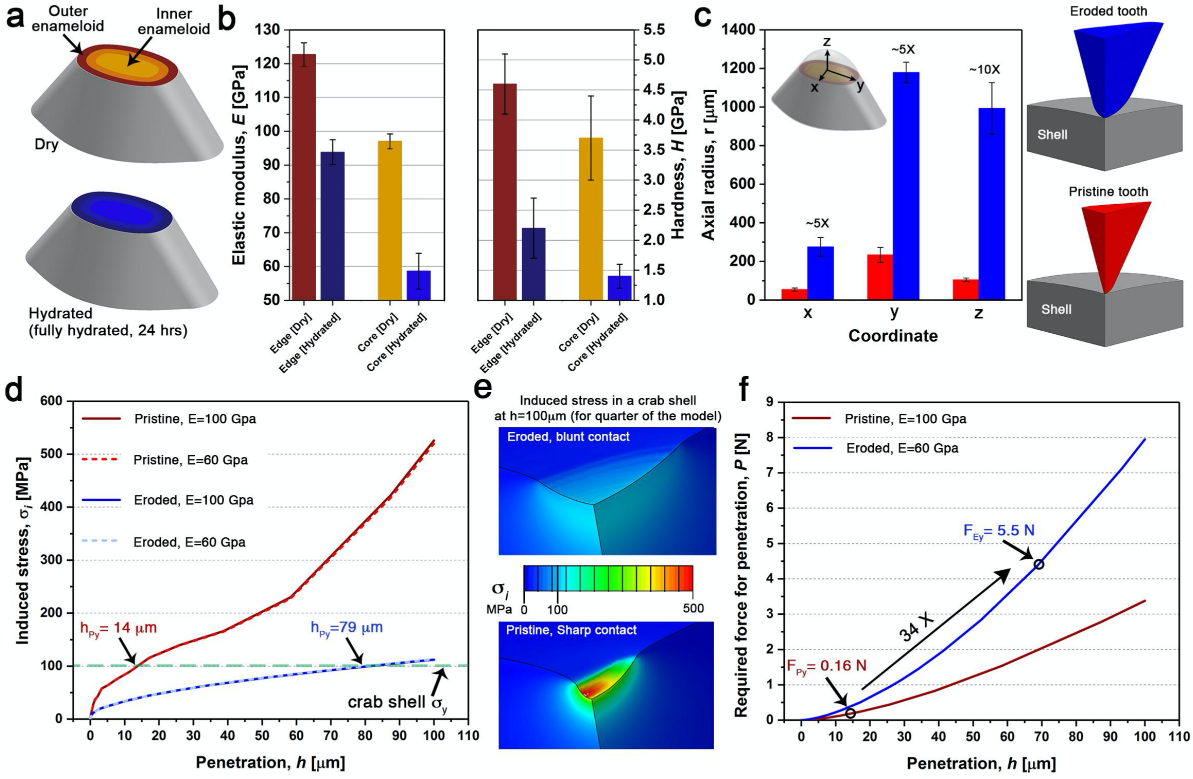

Supplementary Fig. 6 The measured mechanical and geometrical data of the enameloid were used for computational evaluation of the pristine and eroded cusps' performance during contact interactions with a simulated crab shell. **a, b** Nanoindentation studies performed on the outer and inner enameloid in both dry and hydrated conditions revealing a higher elastic modulus and hardness of the outer enameloid (bars represent mean values +/- standard deviations, n>20). **c** The measured radii of the pristine and eroded cusps showing the higher erosion rate in the biting direction (z-axis) (bars represent mean values +/- standard deviations, n=5). **d** Finite element analysis was used to assess the influence of geometry and stiffness on contact properties of pristine and eroded cusp models. Maximum stresses induced in a target (crab shell model) during contact are plotted against the surface displacement depth. For both cusp models, eroded and pristine, two stiffness properties are tested for their influence on that contact stresses (Solid lines: 100 GPa, dashed lines: 60 GPa). **e** Color-coded plots show the von Mises stress distribution at the contact surface of a crab shell model for an eroded and a pristine cusp (top, and bottom, respectively). Stresses are presented at 100 µm penetration depth in a quarter of the model (for symmetry reasons). **f** Reaction forces exerted by the cusp model are plotted against contact surface displacement of the shell, which demonstrates a 34-times larger force is necessary for eroded cusp to realize a penetration in the shell (black circles on the graphs).

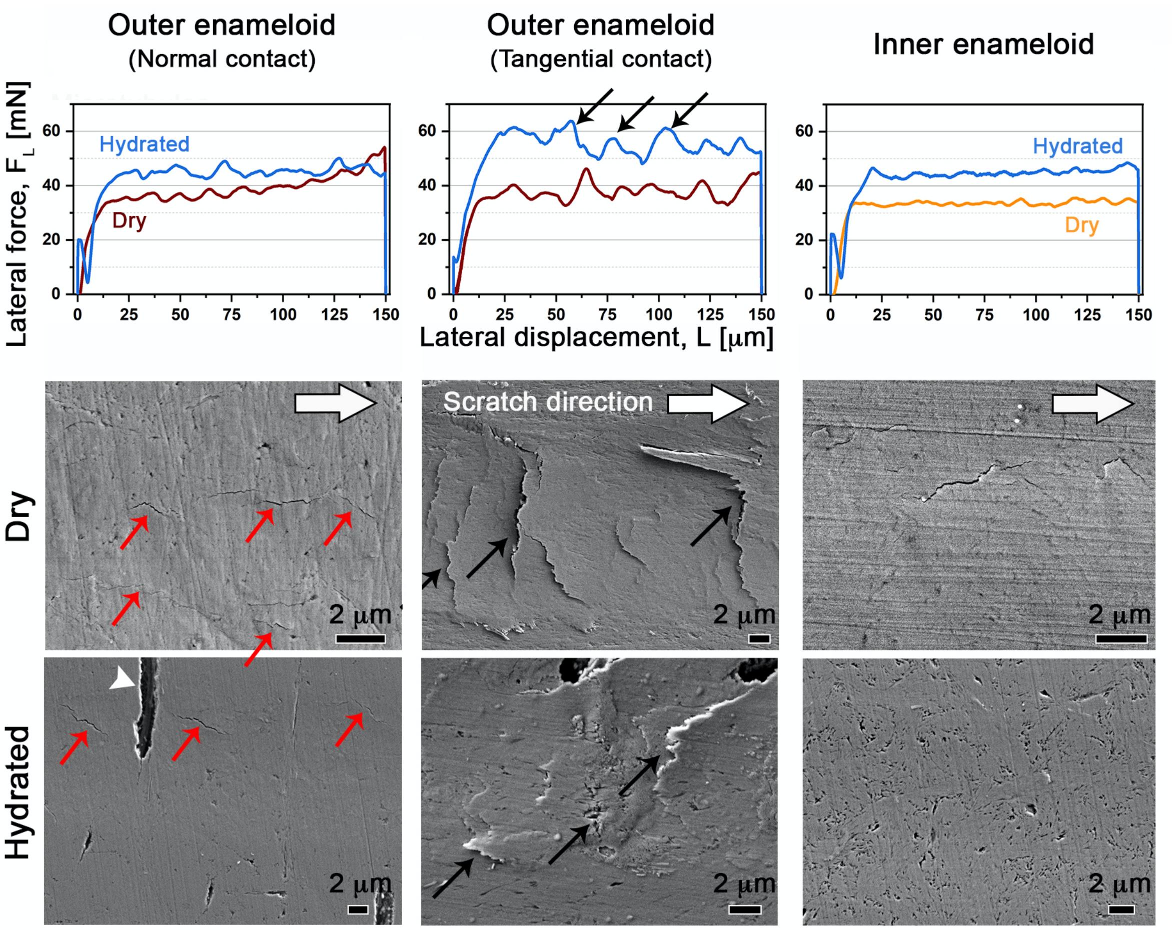

Supplementary Fig. 7 Comparative scratch studies on the different layers and directions of the enameloid layers in hydrated (top) and dry (bottom) conditions. Lateral force-displacement curves show that hydration results in a higher penetration of the tip in the sample, and consequently, the increments in the lateral forces. Post-scratch FESEM micrographs revealing a decrease in crack sizes promoted by a higher strain accommodation (increment in the penetration depths and widening of the scratch tracks). However, the detected damage mechanisms follow similar patterns (Figure 6) in both hydrated and dry condition.

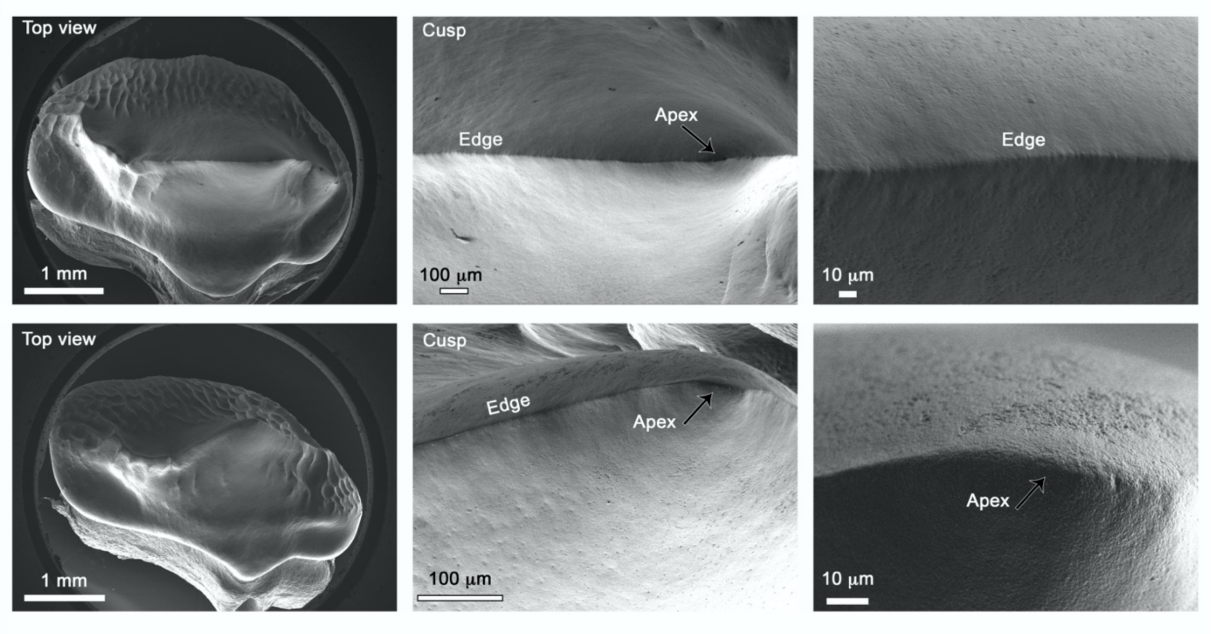

Supplementary Fig. 8 FESEM micrographs captured from the pristine cusps (rows 8 and 9) revealing the absence of cracking in pristine (pre-functional) teeth, underlining that the chipping observed on the functional teeth were induced by mechanical contacts during the teeth function, not a drying artifact.

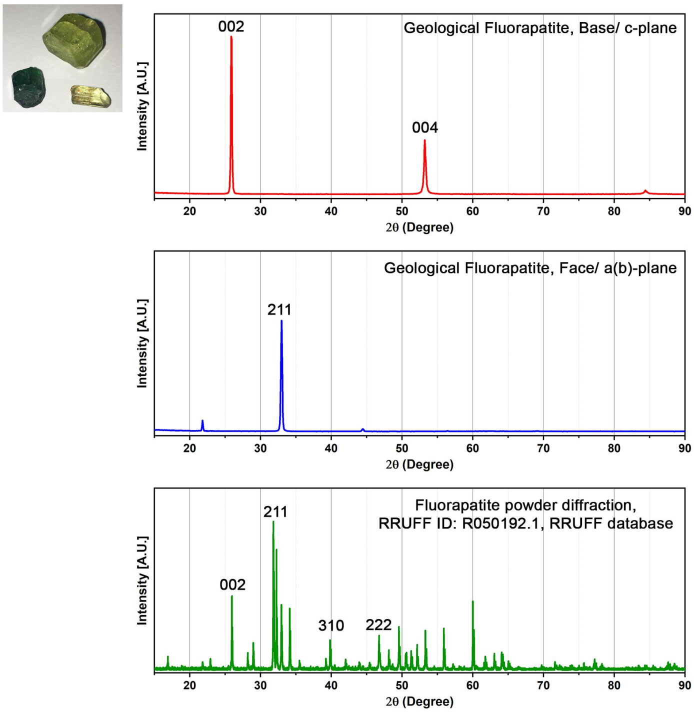

Supplementary Fig. 9 X-ray diffraction pattern of the geological Fluorapatite sample. The obtained peaks from base and facet of the sample revealing the monocrystallinity and confirming the crystallographic orientation of the geological FAP sample.

Supplementary Note 1

Raman crystallography

Since the isolation of individual biological crystallites is difficult, we used measurements of a monocrystal of geological apatite as a standard for determination of the crystallographic orientation of fluorapatite (Supplementary Fig. 9). Accordingly, we measured and collected Raman spectra from the basal and prism facets of the crystal in different orientations (0°, 30°, 45°, 60°, and 90°) using parallel and cross polarizations. By comparing the collected spectra, we could confirm the presence of the 591 cm^{-1} peak and its relative intensity to the 581 cm^{-1} peak in parallel-polarized measurements can elucidate the orientation of the crystals along their c-axis. In addition, using cross-polarized measurements, we were able to differentiate crystallographic planes using the 1059 cm^{-1} peak, which denotes the basal facet of crystal but is absent in spectra of crystal prism facets (Fig. 4b and Supplementary Fig. 5). Using these orientation-dependent guidelines from geological apatite and capitalizing on the high mineral content and low Raman background signal of the shark tooth enameloid, we Raman-mapped a transverse section of the cusp with sub-micron resolution (~300 nm), and filtered the maps for the 581 cm^{-1}, 591 cm^{-1}, and 1059 cm^{-1} peaks, accordingly determining the distribution of the FAP crystallite orientations throughout the enameloid (Fig. 4c).

Supplementary References

1. Leroy G, Leroy N, Penel G, Rey C, Lafforgue P, Bres E. Polarized Micro-Raman Study of Fluorapatite Single Crystals. *Appl Spectrosc* **54**, 1521-1527 (2000).
2. Bentov S*, et al.* Enamel-Like Apatite Crown Covering Amorphous Mineral in a Crayfish Mandible. *Nature Communications* **3**, (2012).
3. Amini S*, et al.* Textured Fluorapatite Bonded to Calcium Sulphate Strengthen Stomatopod Raptorial Appendages. *Nature Communications* **5:3187**, (2014).
